# Individual Differences in Spatial Orientation Modulate Perspective Taking in Listeners

**DOI:** 10.5334/joc.321

**Published:** 2023-09-01

**Authors:** Jia E. Loy, Vera Demberg

**Affiliations:** 1 Department of Language Science and Technology, Saarland University, Germany; 2 Department of Computer Science and Department of Language Science and Technology, Saarland University, Germany

**Keywords:** individual differences, perspective taking, interaction, spatial orientation

## Abstract

Previous research suggests that individuals exhibit consistent tendencies towards taking their own (an egocentric) or their partner’s (an othercentric) spatial perspective. In addition, several factors such as spatial orientation ability, inhibitory control, and social preferences, have been found to mediate these perspective taking tendencies. However, these factors have not been studied together in the context of a single task. The present study explores these individual differences together in spatial perspective taking, using a task of simulated interaction in which listeners can choose to interpret an ambiguous spatial utterance egocentrically or othercentrically. We use a data-driven approach of latent profile analysis to classify participants into subgroups based on their spatial perspective taking tendencies. Our results show that stable subgroups of participants can be identified who differ in their perspective taking tendencies. This behaviour also correlates with a measure of listeners’ spatial orientation ability, but not their inhibitory control or social preferences. Our results can be interpreted within a framework that views spatial perspective taking as an embodied cognitive process of a mental reorientation of the self relative to the environment, providing insight on the nature of the mechanisms underlying this operation.

## Introduction

In everyday action and communication, humans often have to take on a different perspective to their own, for instance when conceptualising or describing objects from another person’s point of view. Such perspective taking relies on the ability to perceive some aspect of our environment in the way it appears to others. Importantly, individuals also demonstrate natural preferences in terms of which perspective they tend to adopt. For instance, when describing the spatial relation between objects from a third person’s point of view, speakers are highly consistent with respect to which frame of reference they use – whether they assume a perspective rooted in an object, a third person, or a superordinate bird’s eye view (Beller, Bohlen, Hüther, & Bender, 2016; Wilke, Bender, & Beller, 2019). When asked to give directions from a map to an imaginary person, speakers tend to show a preference for cardinal directions (north, south, east, or west) or relational terms (left or right), but not both (Ward, Newcombe, & Overton, 1986).

Such tendencies have also been noted in the comprehension of spatial expressions. For instance, Duran, Dale, and Kreuz (2011) investigated spatial perspective taking in listeners using a computerised task in which participants heard instructions from a partner requesting for one of two identical objects. Critically, the instructions made use of the terms *right, left, front*, or *back*, which were potentially ambiguous depending on the seating configuration of the participant and partner around a table (e.g., ‘give me the folder on the right’ could refer to either folder when the participant and partner were seated at opposite sides of the table). Duran et al. determined that listeners could be classified into three groups based on their dominant mode of response – ‘egocentric’ responders, who interpreted the instruction from their own perspective on over 70% of trials; ‘othercentric’ responders, who interpreted the instruction from their partner’s perspective on over 70% of trials; and ‘mixed’ responders who did not show a dominant response preference. Notably, the distribution of participants was largely bimodal, with a roughly even split between egocentric and othercentric responders, and mixed responders in the minority.

Duran et al.’s finding of distinct responder groups reflects systematic variation in listeners’ perspective taking tendencies. However their study falls short of identifying which underlying factors can be attributed to this variability. Moreover, group membership in their study was established via manual thresholds on participants’ response patterns set post-hoc. Thus, it remains unclear whether such grouping of participants is justified based on the actual distribution of the data. These are the questions that the current study is interested in. We investigate inter-individual variation in listeners’ spatial perspective taking behaviour, looking at whether we can observe distinct groups based on a data-driven approach of clustering participants, as well as how various individual difference measures contribute to participants’ perspective taking behaviour. We focus on two broad categories of individual differences which the perspective taking literature has identified: cognitive abilities (e.g., Gardner, Brazier, Edmonds, & Gronholm, 2013; Hegarty & Waller, 2004) and social preferences (e.g., Job, Kirsch, Inard, Arnold, & Auvray, 2021; Shelton, Clements-Stephens, Lam, Pak, & Murray, 2012). In the following sections, we first explain the distinction between different forms of perspective taking and how our study focuses on level-2 spatial perspective taking. We then provide a review of studies that have examined the role of individual differences on spatial perspective taking behaviour.

### Visual and spatial perspective taking

The literature on perspective taking has identified two forms of the process: visual and spatial perspective taking (e.g., Surtees, Apperly, & Samson, 2013a). Visual perspective taking deals with the *visual perception* of a target from another person’s perspective. In particular, a distinction is sometimes made between level-1 and level-2 visual perspective taking. Level-1 visual perspective taking involves a computation of visual accessibility, that is, understanding whether or not a target object can be seen by another person. This form of perspective taking is known to emerge early in development (Flavell, Everett, Croft, & Flavell, 1981; Moll & Tomasello, 2006), and thought to rely on low-level, automatic cognitive resources (Apperly & Butterfill, 2009; Surtees & Apperly, 2012). Level-2 visual perspective taking involves understanding of *how* an object appears visually to another person, and in contrast to level-1 perspective taking, is acquired later in development (Flavell et al., 1981), and theorised to rely on more complex, non-automatic processes (Surtees, Butterfill, & Apperly, 2012).

In contrast to visual perspective taking, spatial perspective taking is concerned with *calculating the location* of objects from another person’s perspective, and relies on reasoning spatially about where something lies in relation to other individuals and objects (e.g., understanding that a ball is to the right rather than left of a chair; Surtees et al., 2013a). As with visual perspective taking, some studies on spatial perspective taking distinguish between two levels of the process. Level-1 spatial perspective taking refers to judgements of whether something lies in front of or behind a target, whereas level-2 spatial perspective taking refers to judgements of whether something lies to the right or to the left (Muto, Matsushita, & Morikawa, 2019; Surtees et al., 2013a). This distinction is motivated by work demonstrating that similar to visual perspective taking, there is a developmental trajectory whereby spatial relations for ‘in front of’ and ‘behind’ are acquired earlier than for ‘right’ and ‘left’ (Harris & Strommen, 1972), and that different computational processes are employed in the two (Surtees et al., 2013a). Judging whether something lies to the right or left of someone else is theorised to require mentally rotating into that person’s perspective, whereas judging whether something is in front of or behind someone does not necessarily demand updating of mental representations, but may rely on visibility-related cues, such as a person’s face (cf. Muto et al., 2019). There is additionally some inconsistency in the classification employed by researchers. For example Kessler and Rutherford (2010) do not separate visual and spatial perspective taking, but rather consider visuospatial perspective taking as a whole, and distinguish between level-1 visuospatial perspective taking (knowledge regarding an object’s visual accessibility), and level-2 (mentally adopting another person’s perspective). In a similar vein, Michelon and Zacks (2006) concern themselves with two levels of visual perspective taking, but their level-2 judgement task (determining whether an object was to the right or left of an avatar) was more akin to a level-2 spatial perspective judgement (cf. Kessler & Wang, 2012). Whilst we are not concerned with differentiating between the different types and levels of perspective taking, for consistency with the recent spatial perspective literature, we adopt the classification employed by Surtees and colleagues which differentiates between level-1 and level-2 spatial perspective taking.

In the current study, we are concerned with level-2 spatial perspective taking, in which listeners adopt a different perspective via having to mentally rotate into a partner’s perspective rather than visibility cues. Specifically, listeners hear ambiguous utterances produced by a partner in which the spatial terms “right”, “left”, “front”, and “back” can refer to different objects depending on whether they adopt their own or their partner’s point of view. We note that the front/back distinction here differs from the in front of/behind dimension in level-1 spatial perspective taking – here, both front and back objects are still within the speaker’s field-of-vision, and therefore presumed to call upon a level-2 mental rotation mechanism.

### Individual differences in cognitive ability

#### Spatial orientation ability

Cognitive strategies linked to spatial perspective taking are theorised to involve an element of mental spatial transformation. This is typically conceptualised as the ability to rotate images or objects in one’s mind. Correspondingly, a number of studies build on the fundamental assumption of a link between object-based rotation and spatial perspective taking. However, results from these studies are mixed, with some studies demonstrating that object-based rotation abilities correlate with perspective taking in individuals (e.g., Menchaca-Brandan, Liu, Oman, & Natapoff, 2007; Muto, 2021), while others fail to replicate this pattern (e.g., Samson, Apperly, Kathirgamanathan, & Humphreys, 2005). The latter finding supports a view that distinguishes between object-based rotation and perspective rotation. While the two have superficially equivalent outcomes (e.g., rotating an array clockwise has the same outcome as the viewer rotating themselves anti-clockwise around the array), they invoke cognitively distinct processes. Specifically, object-based rotations involve mentally rotating an external object relative to the self or the environment, while perspective rotations are seen as an embodied cognitive process of mentally (re)orienting one’s self (i.e. motor simulation of whole-body movement; Muto, Matsushita, & Morikawa, 2018; Surtees et al., 2013a; Yeh, Wang, Cheng, & Chiu, 2021). Level-2 spatial perspective taking in particular has been theorised to require a rotation of one’s mental representation of their body to align itself with the axis of the target perspective (Muto, 2021; Surtees et al., 2013a). Early evidence for dissociation of the two comes from studies which show that performance differs for object-based and perspective rotation tasks with respect to pattern of errors (Huttenlocher & Presson, 1973; Inagaki et al., 2002) as well as response times (Shepard & Metzler, 1971). The latter tend to increase linearly with the degree of angular disparity in object-based rotation, whereas the relationship between perspective rotation and effort appears more complex, with speed and accuracy typically poorest at 180° (suggesting similar strategies for 90° and 270° rotations). Perspective rotation is also known to be influenced by additional factors such as the simplicity of the array (e.g., single vs. multiple objects) and body posture congruency with the target perspective (Kessler & Rutherford, 2010; Surtees, Apperly, & Samson, 2013b; Wraga, Creem, & Proffitt, 2000). A recent study by Muto et al. (2019) additionally highlights the relevance of the reference object’s symmetry: by manipulating the symmetry plane of the reference object (right/left, or front/back), they showed that perspective rotation tends to be elicited for judgements orthogonal to the object’s symmetrical plane (e.g., right/left judgements for a right-left symmetrical object), but not for judgements along the object’s symmetrical plane (e.g., in front of/behind judgements for a right-left symmetrical object).

As a direct investigation of the mechanism underlying perspective rotation, Hegarty and colleagues developed the Object Perspective Test (OPT), a psychometric test of spatial orientation designed to tap into the ability to perform mental rotations of the self (Hegarty & Waller, 2004; Kozhevnikov & Hegarty, 2001). In the task, participants are shown a two-dimensional array of objects and asked to make a judgement of relative direction from an adopted perspective within the array (e.g., ‘Imagine you are at the stop sign and facing the house. Point to the traffic light’). Confirmatory factor analyses showed that the measure derived from the OPT loaded onto the same factor as other tests known to rely on egocentric perspective rotation (e.g., the Money Road Map Test; Money, Alexander, & Walker, 1965), and separate from a factor corresponding to tasks that use object-based rotation (e.g., the Vandenberg Mental Rotation Test; Vandenberg & Kuse, 1978). In addition, the dominant strategy reported by participants post-hoc was to imagine themselves reoriented within the array, as opposed to, for instance, mentally rotating the array (Kozhevnikov & Hegarty, 2001). The results of Hegarty and colleagues highlight the dissociation between object-based rotation (mentally rotating an object) and perspective rotation (reorientation of the ‘self’), and provide evidence for the OPT as a valid test of the latter ability.

Since its conceptualisation, the OPT has been used extensively in the spatial cognition literature, in particular as an indicator of individual differences in spatial orientation. For instance, performance in the OPT has been shown to correlate with how well participants are able to navigate routes in virtual reality (Galati, Weisberg, Newcombe, & Avraamides, 2015; Weisberg, Schinazi, Newcombe, Shipley, & Epstein, 2014), learn spatial layouts from a map (Schinazi, Nardi, Newcombe, Shipley, & Epstein, 2013), and reproduce aerial models of a learned environment (Weisberg et al., 2014). Notably, these tasks all involve an element of updating a mental representation of oneself in space, a crucial component of an imagined change in perspective (cf. May, 2004).

#### Inhibitory control

Perspective taking (both visual and spatial) has also been linked to higher-level cognitive functions such as inhibitory control. Underlying the view that perspective taking involves an effortful mental updating of one’s self-representation is the implication that it requires inhibiting one’s current perspective in order to align with the imagined target perspective. Indeed, several studies highlight a relationship between inhibitory control and perspective taking. The majority of these studies make use of paradigms that test participants’ ability to track whether or how objects appear to a partner, and thus largely remain within the domain of visual perspective taking (e.g., Brown-Schmidt, 2009; Qureshi, Apperly, & Samson, 2010; Wardlow, 2013). However, a small number of studies have employed tasks of spatial perspective taking, and demonstrate a relationship with inhibitory control, in both adults (Gardner et al., 2013) as well as children (Frick & Baumeler, 2017). For instance, Gardner et al. (2013) found that participants’ response times in determining whether a ball was in the left or right hand of an avatar were related to their performance in a control task of response inhibition, although the finding was limited to the subset of participants who self-reported having adopted a “spatial transposing” strategy (i.e. transposing “left” and “right” when the avatar was facing them).

While a range of tests are used to measure inhibition, one commonly used test is the Stroop task, in which participants have to attend to one feature of a stimulus whilst ignoring another, more dominant, feature (e.g., saying ‘green’ when the word *green* is presented in red text; Stroop, 1935). Similar variants (e.g., the Fruit Stroop task) are often used with developmental populations (e.g., Frick & Baumeler, 2017). Lower inhibitory control, as measured by poorer performance on the task, is typically associated with poorer perspective taking. The ability to adopt a third-person’s visual perspective is also negatively affected when performed alongside a secondary task that requires inhibiting a prepotent response (e.g., Luria’s tapping task; Diamond & Taylor, 1996), suggesting that inhibition is a process common to the two tasks (Qureshi et al., 2010). Although the relationship between inhibition and spatial perspective taking is less well-established, a common finding within the spatial cognition literature highlights the primacy of an egocentric perspective (e.g. Shelton & McNamara, 2001; Yadollahi, Couto, Dillenbourg, & Paiva, 2022), suggesting a certain degree of effort necessary in suppressing this to adopt an othercentric perspective. Going beyond spatial representations, the notion of an egocentric primacy is also consistent with broader findings demonstrating the role of inhibitory processes in other domains such as language, beliefs, and reasoning (Brookman-Byrne, Mareschal, Tolmie, & Dumontheil, 2018; Epley, Keysar, Van Boven, & Gilovich, 2004; Horton & Keysar, 1996; Zhang, Sha, Zheng, Ouyang, & Li, 2009).

### Individual differences in social preferences

A separate line of research focuses on the social dimension of spatial perspective taking. Some researchers characterise perspective taking as an automatic social process driven by attempts to decipher another’s point-of-view in anticipation of socially-relevant functions, such as interaction (e.g., Clements-Stephens, Vasiljevic, Murray, & Shelton, 2013; Tversky & Hard, 2009; Xiao, Xu, Sui, & Zhou, 2021). This account is based on the view that humans are inherently social beings who evoke perspective taking as a strategy to effectively navigate social and communicative situations. Indeed, the capacity to understand another person’s perspective is an ability central to human cognition, and one that is much less developed in other species (cf. Tomasello 2009). Tversky and Hard (2009) demonstrate the role of social cognition in perspective taking through an experiment that manipulated the presence of a person within a scene on participants’ spatial descriptions: simply the presence of another person (even when he did not interact with any objects in the scene) led to an increase in participants’ tendencies to produce descriptions from that person’s perspective rather than an egocentric perspective. Drawing attention to an action performed by the person through the phrasing of a question (e.g., ‘In relation to the bottle, where *does he place* the book?’ [italics added here for emphasis]) further increased participants’ othercentric descriptions. The authors interpret this as evidence that perspective taking could be a spontaneous behaviour that arises in response to a potentially social situation.

Building on this view, several researchers have gone on to demonstrate a relationship between spatial perspective taking and an individual’s social preferences (Job et al., 2021; Kessler & Wang, 2012; Shelton et al., 2012). Here, it is worth noting that researchers have used a range of measures to define and assess social preferences. Using the Autism Quotient (AQ; Baron-Cohen, Wheelwright, Skinner, Martin, & Clubley, 2001), Shelton et al. (2012) found that participants with better social skills (as quantified by their score on the social skills and communication subscales of the AQ) were also better able to recognise the perspective that an observer had of a three-dimensional display (the Three Mountains task; cf. Piaget & Inhelder, 1967). Importantly, this relationship only held when the ‘observer’ was represented by a wooden human figure, and not when it was a camera or triangular block, highlighting the importance of social agency in spatial perspective taking (cf. Xiao et al., 2021). Kessler and Wang (2012) also demonstrate the relevance of the social skills subscale of the AQ, such that differences in this measure correlate with different strategies in spatial perspective taking: participants with higher social skills appeared to exhibit stronger embodiment effects when adopting another perspective. A related measure of social intelligence – the ability to process social information – has also been shown to modulate perspective taking abilities: individuals with higher social intelligence, as measured by the Tromsø Social Intelligence Scale (TSIS; Silvera, Martinussen, & Dahl, 2001), show a greater improvement in performance when switching from an unnatural, instructed perspective to their own natural perspective (Job et al., 2021). Regardless of the measure employed, the general finding is that weaker social preferences tend to be associated with poorer perspective taking. Complementary evidence from other aspects of social functioning support this view. Erle and Topolinski (2015) for instance, highlight a degree of commonality in the mechanisms underlying empathy and spatial perspective taking. In their study, better performance in a spatial task of judging whether an object was on the right or left of an avatar (cf. Kessler & Wang, 2012) was correlated with higher levels of self-reported empathic perspective taking (as measured by Perspective-Taking scale on the German version of the Interpersonal Reactivity Index (IRI; Paulus, 2009)). Finally, research on Autism Spectrum Disorders (ASDs) also points to a relationship between social impairments, such as difficulty with theory of mind, and poorer perspective taking ability (e.g., Hamilton, Brindley, & Frith, 2009), further highlighting the general link between social preferences and perspective taking.

### Current study

The current study explores individual differences in listeners’ spatial perspective taking behaviour. We focus on individual difference measures in cognitive abilities (spatial orientation ability and inhibitory control) and social preferences. Previous studies have demonstrated the role of each of these individual differences on perspective taking; however, these three factors have thus far not been investigated together. This is a potential limitation since the contribution of factors on an outcome can change depending on which factors are included in the analysis; in only taking one factor into consideration, researchers may overestimate its contribution or overlook the contribution of other, more directly relevant factors (Freed, Hamilton, & Long, 2017). Thus, in taking all three factors into account, we provide a more holistic picture of the relative contribution of various individual differences measures on spatial perspective taking behaviour. Moreover, the majority of existing studies focus on perspective taking behaviour in speakers, or else in comprehenders instructed to explicitly adopt a particular perspective. Here, we are interested in how individual differences may modulate perspective *choice* in comprehenders, i.e. whether they opt to take their own or the speaker’s perspective. This is an important question to ask since real-life spatial reasoning can often involve ambiguity (e.g., do you mean my left or your left?), thus invoking an element of choice in listeners’ behaviour. Previous work by Duran et al. (2011) shows that considerable inter-individual variation exists in listeners’ perspective choice tendencies, resulting in distinct categories of participants (‘egocentric’, ‘othercentric’, ‘mixed’). However, their study used manual thresholds set post-hoc to establish category membership in listeners. In the current study, we adopt a data-driven method of classifying listeners via Latent Profile Analysis (LPA) to determine whether such sub-groups emerge in the data.

Thus, in the current study we aim to answer the following questions:

1. Can we identify latent groups of participants based on their spatial perspective taking tendencies?
2. Which individual differences measures contribute to spatial perspective taking behaviour?
3. How do latent groups differ in these individual difference measures?

The main spatial perspective taking task in the study was modelled on Duran et al.’s (2011) experiment, in which participants manipulate objects on a table top following a partner’s directions (e.g., ‘give me the potato on the right/left/front/back’), which could be interpreted egocentrically or othercentrically. Following which, participants completed an individual differences battery which consisted of the following tests: the Autism Quotient (AQ; Baron-Cohen et al., 2001), the Object Perspective Test (OPT; Hegarty & Waller, 2004; Kozhevnikov & Hegarty, 2001), and the colour-word Stroop task (Stroop, 1935).^1^

## Method

### Participants

Two-hundred and ninety-one participants were recruited on AMT (AMT).^2^ We used AMT filters to restrict recruitment to US-based participants with a minimum of 1000 approved HITs and a 97% approval rating. The study took approximately 35 minutes and participants received US$7 compensation for completing the full set of tasks.

For the analyses, we excluded data from participants who: (a) reported they were non-native speakers of English in the post-test questionnaire (2 participants), and/or (b) failed to meet a minimum accuracy threshold of 80% on all trials except for those in the different perspective condition in which the partner’s utterance was ambiguous (36 participants). An additional 73 participants who either dropped out part-way through the individual differences battery or failed to meet criteria on the Stroop task (>90% accuracy) were also excluded. In addition, it emerged post-hoc that a subset of participants had confused the terms “front” and “back” by interpreting these from a top-down view rather than from the avatar’s perspective (see Galati, Dale, & Duran, 2019 for a related discussion), thus mapping “front” to the top of the screen and “back” to the bottom (i.e. the opposite outcome to what was intended). Thus, as a conservative measure, we also excluded participants who selected the wrong object on more than one same perspective trial (21).^3^ Hence, the final dataset consisted of 159 participants (61 female, 98 male; mean age = 40 years (*SD* = 11, range = 22–72)).

### Materials and design

**Figure 1:**
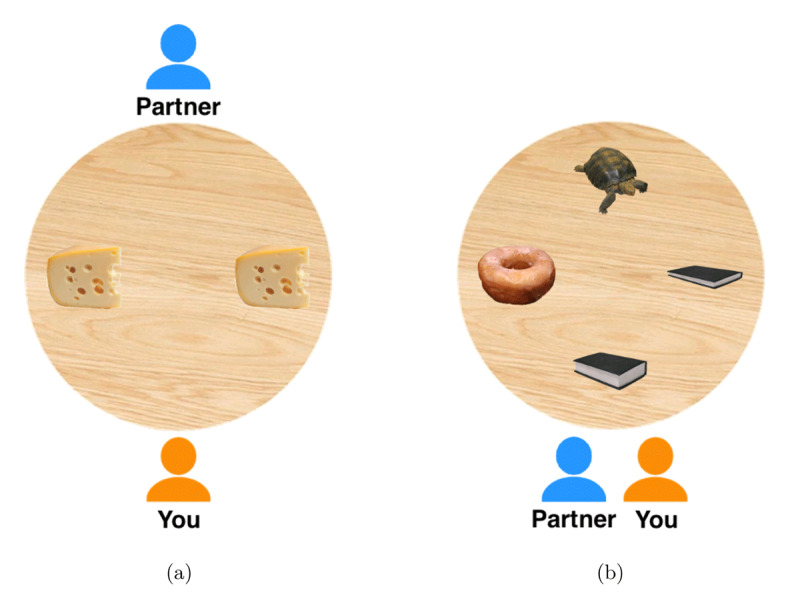
Example of **(a)** a critical trial in the different perspective condition with right/left ambiguity, and **(b)** a filler trial with size contrast (thin/thick book).

The main task of the experiment consisted of 16 critical and 36 filler trials. Each trial presented a number of objects (two, three, or four) arranged on a table top viewed from above. Objects were located in one of four pre-determined positions: top, left, bottom, or right of the centre of the table. Each trial also featured two avatars representing the participant and their partner (orange and blue respectively). The participant was always located at the bottom of the table; we manipulated whether the partner was located next to the participant (same perspective condition) or across the table from the participant (different perspective condition). Figure 1 shows an example of a trial from the task.

Objects used in the experiment were images of everyday objects (e.g., clock, stapler, potato) taken from the Bank of Standardised Stimuli (BOSS; Brodeur, Dionne-Dostie, Montreuil, & Lepage, 2010). In cases where no suitable images were available on BOSS, a free alternative was sourced from Google Image. A total of 64 images were used in the experiment: 32 unique objects and 16 contrast pairs (e.g., red/green apple, long/short pencil). Contrast pairs were created by editing the original image to produce two versions of the object that differed only in the relevant contrast property, and were featured in filler trials (see below).

Critical displays always featured two identical objects. These were located in either the left and right positions, or the top and bottom positions on the table. Eight critical trials used the same perspective seating configuration and the other eight used the different perspective configuration. The partner’s request was always of the form ‘Give me the < object > on the left/right/front/back’. Participants were told to follow instructions from their partner, which was a simulated computer program. Partner instructions were synthesised with the Apple Macintosh built-in text-to-speech function (‘Agnes’ voice). Half of the trials in each perspective condition were front/back utterances and the other half were left/right utterances.

To reduce the salience of critical displays, we included filler trials in which we varied the total number of objects presented (two, three, or four), as well as the type of display. Three types of filler displays were used, in which referent identification involved either: (a) a colour contrast (e.g., red/green apple), (b) a size contrast (e.g., long/short ruler), or (c) no contrast (identifiable by the bare noun alone, e.g., ‘clock’, with unrelated distractors). Each display type was used in 12 filler trials. Half of the trials within each display type used the same perspective seating configuration and the other half used the different perspective configuration. A higher proportion of three- and four-object filler displays were included to ensure participants saw roughly the same number of two-, three- and four-object displays across the experiment. Table 1 provides a breakdown of the variation in display types used in the experiment.

**Table 1:** Breakdown of display types in the experiment.


TRIAL TYPE	DISPLAY TYPE	NO. OF OBJECTS	NO. OF TRIALS

critical	same perspective (left/right)	2	4

	different perspective (left/right)	2	4

	same perspective (front/back)	2	4

	different perspective (front/back)	2	4

filler	colour contrast	2/3/4	12

	size contrast	2/3/4	12

	no contrast	2/3/4	12


Distractor objects on filler trials were chosen randomly from the full set of images with the constraints that (a) any relevant contrast requirements were fulfilled, and (b) no objects were repeated in the display. The position of objects on fillers was randomised, and the partner’s instruction unambiguously identified a single referent (e.g., ‘Give me the red apple’, or ‘Give me the clock’).

### Procedure

Participants accessed the experiment online via the AMT website. The experiment was described as a test of an online interface in which two users carried out a joint task in a shared virtual workspace. The participant’s task was to move objects about the workspace in response to spoken instructions from their partner, who was a simulated computer program. The instructions emphasised that the interface was still in its development stages, hence audio streaming only worked one-way (i.e. participants could hear but could not speak to their partner). Following this, participants were taken to the audio check phase. This phase served to ensure participants had audio turned on and volume adjusted to a suitable level. Participants followed their partner’s instruction to click on a target image out of an array of four images. After selecting the correct image, the task began. Participants who selected the wrong image more than once were prevented from continuing with the experiment.

During the task, participants saw a display consisting of a table top viewed from above and two avatars representing the participant and their partner. On each trial, the display featured a number of objects arranged on the table top. After a short delay, playback of the partner’s instruction began, in which they would request one of the objects. The delay was fixed at 1,200 ms on critical trials, and variable between 800–2000 ms on filler trials (with lower probability assigned to larger values). Participants manipulated objects by clicking on and dragging them over to their partner’s avatar. Objects were not movable until the partner’s utterance had finished playing. Once an object had been ‘given’ to the partner, the objects disappeared and were replaced by the objects for the next trial. A progress bar at the top of the screen indicated how many trials the participant had completed. Trial order was randomised for each participant with the constraints that the task began with at least three filler trials, and critical trials were separated by at least one filler trial.

### Individual differences battery

Following the main task, participants completed an individual differences battery consisting of four tasks in the following order: the AQ, the OPT, the Stroop task, and the direction discrimination task.

#### Autism Quotient

The social skills and communications subscales of the AQ was used as a measure of participants’ social preferences. Participants completed the full AQ – a 50-item self-administered questionnaire designed to assess traits associated with autism spectrum disorders (ASDs) in neurotypical adults (Baron-Cohen et al., 2001). The test consists of statements targeting behavioural characteristics of ASDs (e.g., ‘I would rather go to a library than a party’), which respondents rate on a four-point Likert scale (definitely agree, slightly agree, slightly disagree, definitely disagree). The test measures five traits: social skills, communication, attention to detail, attention switching, and imagination, with each subscale based on ten questions. In our analyses, we focused on the social skills and communication subscales, which we take as a proxy for social preferences (cf. (Shelton et al., 2012; Stewart & Austin, 2009). A combined score based on those two subscales was derived for each participant (AQ_ss+c_). We employed Austin’s (2005) scoring strategy of assigning 1–4 to values of the scale, using reverse keying when necessary. The final measure was a score ranging from 20–80 for each participant, with higher scores reflecting weaker social preferences.

#### Object Perspective Test

**Figure 2:**
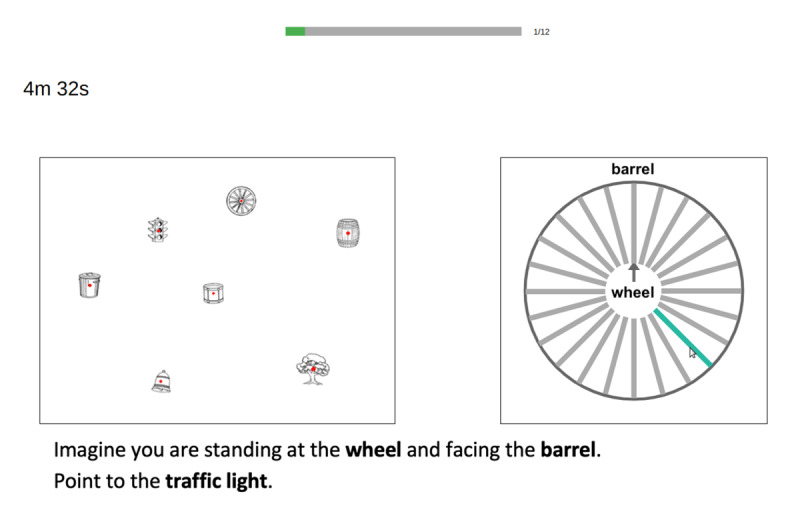
Example of the display participants saw in the OPT. Participants indicated their response by clicking on one of the 24 lines within the response circle, which would be highlighted when their mouse hovered over it.

A computerised version of the OPT developed by Kozhevnikov and Hegarty (2001; later refined by Hegarty & Waller, 2004) was used as a measure of participants’ spatial orientation ability. The test is a task of visualisation designed to tap into the ability to perform mental orientations of the self relative to the environment. On each trial, participants see a configuration of seven objects, and are asked to imagine themselves located at one of the objects (the *station point*) and facing another object (the *heading*), and then indicate the direction from that perspective to a third object (the *target*). We used the array developed by Friedman, Kohler, Gunalp, Boone, and Hegarty (2020, Experiment 2), which replaced stimuli in the original test with a set of inanimate, non-directional objects to address issues of directionality associated with the original set. The task consisted of 12 trials. The array remained the same on each trial, while the station, heading, and target objects changed from trial to trial. Participants responded by clicking on one of 24 lines spaced at 15° increments within a response circle (see Figure 2). All trials required a perspective change of more than 90° (cf. Hegarty & Waller, 2004). As in the original OPT, participants had five minutes to complete the test; a timer on the screen indicated how much time the participant had left. After responding on each trial, the task automatically moved on to the next trial with no possibility of returning to previous trials. Trials were scored by taking the absolute angle deviation in degrees between the participant’s response and the correct answer. A final score for each participant was calculated by taking the average deviation across all 12 trials, with higher scores indicating poorer spatial orientation.

#### Stroop task

The colour-word Stroop task was used as a measure of participants’ inhibitory control. The test taps into the ability to attend to one feature of a stimulus while ignoring interference from another feature (Stroop, 1935). In this version of the test, on each trial participants saw a colour word (‘red’, ‘blue’, ‘green’, ‘yellow’, or ‘purple’) which would be presented in one of the five colours. On congruent trials, the word matched the colour of the text; on incongruent trials the word differed from the colour of the text (Hedge, Powell, & Sumner, 2018). Participants responded by pressing the key corresponding to the first letter of the colour of the text (r, b, g, y, p); thus the task required participants to attend to the colour of the text whilst ignoring the word. After responding, participants were shown feedback (correct/wrong) for 500 ms before the next trial began. Participants completed a total of 100 trials—60 congruent and 40 incongruent, presented in random order. Each word appeared 12 times in the congruent condition and eight times in the incongruent condition (twice in each of the other four colours). A Stroop effect for each participant was calculated by taking the difference between their mean response time in the incongruent and congruent conditions, with a greater Stroop effect (larger difference) reflective of poorer inhibitory control. Data from participants whose overall accuracy was <90% were excluded from analyses (cf. Hasshim & Parris, 2021).

## Analyses and Results

We first coded data from the main task for spatial perspective taking behaviour. This was derived from the measure of whether or not participants selected the object from their own avatar’s perspective (e.g., the book on the left when viewed from their avatar in response to the utterance “give me the book on the left”). On same perspective trials this was always a shared perspective with the partner’s avatar, hence we would own-avatar object selection to be at ceiling. Of interest is participants’ object selection on different perspective trials, where selection of the object from their own avatar’s perspective reflects egocentric perspective taking. For each participant, we also calculated their rate of egocentricism as a proportion out of the total number of different perspective critical trials.

Statistical analyses were carried out in R version 4.1.0 (R Core Team, 2021). Our analyses aimed to answer the three main questions: (a) whether there are latent groups of participants who differ in their spatial perspective taking tendencies, (b) which individual differences measures contribute to spatial perspective taking behaviour, and (c) how latent groups differ in their individual differences measures. In this section, we first provide an overview of the data in the main task, followed by the analyses addressing each question. We also provide an overview of the descriptive statistics and distributions of our three individual differences measures in Appendix A (Figure 8; Tables 5, 6, 7, 8).

**Figure 3:**
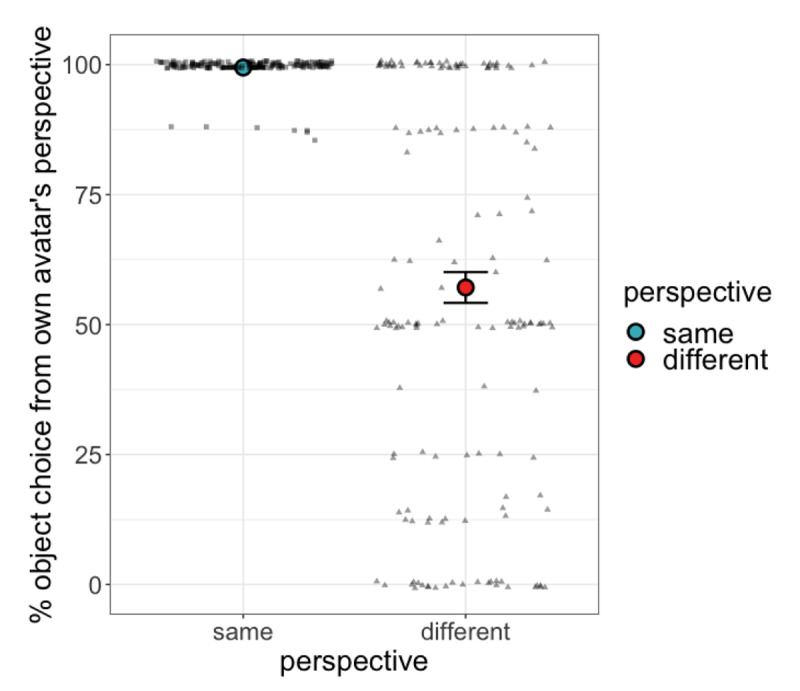
Percentage of trials on which participants chose the object from their own avatar’s perspective (on same perspective trials this was a shared perspective with the partner’s avatar; on different perspective trials this was across the table from the partner’s avatar and therefore reflects egocentric perspective taking by the participant). Error bars represent ±1 SE of by-participant means. Dots represent individual participants.

The final dataset consisted of data from 159 participants who each contributed 16 data points (eight same perspective; eight different perspective trials). Figure 3 shows the percentage of trials on which participants selected the object from the perspective of their own avatar. Of interest is participants’ behaviour on different perspective trials, where this response reflects egocentric perspective taking by the participant.

To verify that our perspective manipulation had the intended effect (i.e. that participants did take their partner’s perspective on different perspective trials), we ran a logistic mixed effects regression to model the dependent variable of whether or not participants selected the object from their own avatar’s perspective on each trial. Of interest here is their behaviour on different perspective trials, where own-avatar object selection reflects egocentric perspective taking. We included perspective (sum coded with levels –0.5 for same and 0.5 for different) as a predictor. Since participant age or gender could also influence perspective taking, these were added as co-variates to the model by including their respective interactions with perspective. The model included by-participant and by-item random intercepts and by-participant random slopes for perspective.

The model showed an effect of perspective, with participants less likely to select the own-avatar associated object on different perspective trials, *β =* –4.54, *p* < .001, *CI* [–5.44, –3.64]. This confirms that when both egocentric and othercentric interpretations were valid, participants responded othercentrically more often than when only an egocentric interpretation was valid; in other words, participants took their partner’s perspective at least some of the time when the opportunity was present. Neither age nor gender showed an interaction with perspective (all *p* > .2). Notably, we also observed considerable individual variation in participants’ egocentric tendencies. This is apparent when looking at the dispersion of individual participants in the different perspective condition in Figure 3. Participants were broadly distributed with respect to their response in the different perspective condition, with larger concentrations of participants whose behaviour was fully egocentric, fully othercentric, and precisely in between. This suggests that many listeners exhibit consistent tendencies in their spatial perspective taking behaviour, lending support to a classification of participants based on this behaviour.

### Latent participant groups

To address our first question of whether classification of participants into groups is supported by the data, we ran a Latent Profile Analysis (LPA) using the tidyLPA package (Rosenberg, Beymer, Anderson, Van Lissa, & Schmidt, 2018) in R. Previous work has used manually-determined thresholds to classify participants into groups based on their egocentric tendencies (e.g., Duran et al., 2011). Here, we use a data-driven approach to identify subgroups in an unclassified sample, which allows us to examine whether such groups can be observed without using a priori classification of participants.

Participants’ rate of egocentricism was used as input to the models. A model selection approach was taken to estimate the optimal number of groups, comparing model solutions from one to five groups (a six group solution could not be estimated). Each model was run 100 times to avoid local maxima. In selecting a final model, Weller, Bowen, and Faubert (2020) recommend taking into consideration several fit statistics alongside interpretability of the model itself. Hence, we assessed model fit using common fit measures, including Akaike’s Information Criterion (AIC; Akaike, 1998) and Bayesian Information Criterion (BIC; Schwarz, 1978), as well as a weighted solution, the composite relative importance vector (C-RIV) from the Analytic Hierarchy Process (Akogul & Erisoglu, 2017). This vector is derived from the pairwise comparison matrix of several fit indices, namely AIC, BIC, Approximate Weight of Evidence (AWE; Banfield & Raftery, 1993), Classification Likelihood Criterion (CLC; Biernacki & Govaert, 1997), and Kullback Information Criterion (KIC; Cavanaugh, 1999). Higher C-RIV values are taken as evidence for the preferred number of groups (see Akogul & Erisoglu, 2017 for a detailed explanation). In addition, we considered the interpretability of classification profiles, as well as global fit statistics such as model entropy (e.g., Kapantzoglou, Restrepo, Gray, & Thompson, 2015).

Table 2 provides a summary of the classification indices and global fit statistics of the models we compared. The AIC and BIC values showed a predominantly downward trend, and both measures posit the five-group model as the best solution. The C-RIV from the AHP comparing one- to five-group solutions favoured the three-group model as the best solution. With the exception of a one-group model, Model 3 also had the highest entropy estimate, suggesting the best separation of groups and assignment of individuals to a particular group (M.-C. Wang, Deng, Bi, Ye, & Yang, 2017).

**Table 2:** Classification indices and fit statistics for LPA models.


MODEL	GROUP DESCRIPTION	RATE (%)	% OF SAMPLE	AIC	BIC	ENTROPY	LOGLIK	C-CIV

**Model 1**				141.45	147.58	1.0	–68.72	0.180

— Group 1	NA	0.0 – 100.0	100.0					

**Model 2**				111.43	123.70	0.73	–51.71	0.219

— Group 1	Mixed (more othercentric)	0.0 – <50.0	47.8					

— Group 2	Mixed (more egocentric)	50.0 – 100.0	52.2					

**Model 3**				–3.88	14.53	0.98	7.94	5.05

— Group 1	Othercentric	0.0 – <37.5	29.6					

— Group 2	Mixed	37.5 – <75	30.2					

— Group 3	Egocentric	75 – 100.0	40.3					

**Model 4**				–17.34	7.21	0.94	16.67	–3.20

— Group 1	Othercentric	0.0 – <12.5	15.7					

— Group 2	Mixed (more othercentric)	12.5 – <37.5	13.8					

— Group 3	Mixed	37.5 – <75.0	30.2					

— Group 4	Egocentric	75.0 – 100.0	40.3					

**Model 5**				–38.20	–7.51	0.97	29.10	–1.24

— Group 1	Othercentric	0 – <12.5	15.7					

— Group 2	Mixed (more othercentric)	12.5 – <37.5	13.8					

— Group 3	Mixed	24.5 – <62.5	30.6					

— Group 4	Mixed (more egocentric)	62.5 – <83.3	6.3					

— Group 5	Egocentric	83.3 – 100.0	39.6					


Notes: Rate = range of rate of egocentricism for group; AIC = Akaike Information Criterion; BIC = Bayesian Information Criterion; logLik = log likelihood of model; C-RIV = Composite Relative Importance Vector.

**Figure 4:**
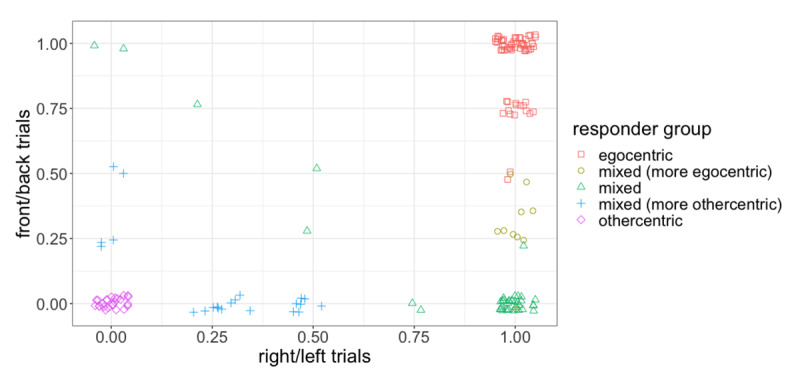
Mean rate of egocentricism on front/back and right/left trials. Each point represents a single participant.

**Figure 5:**
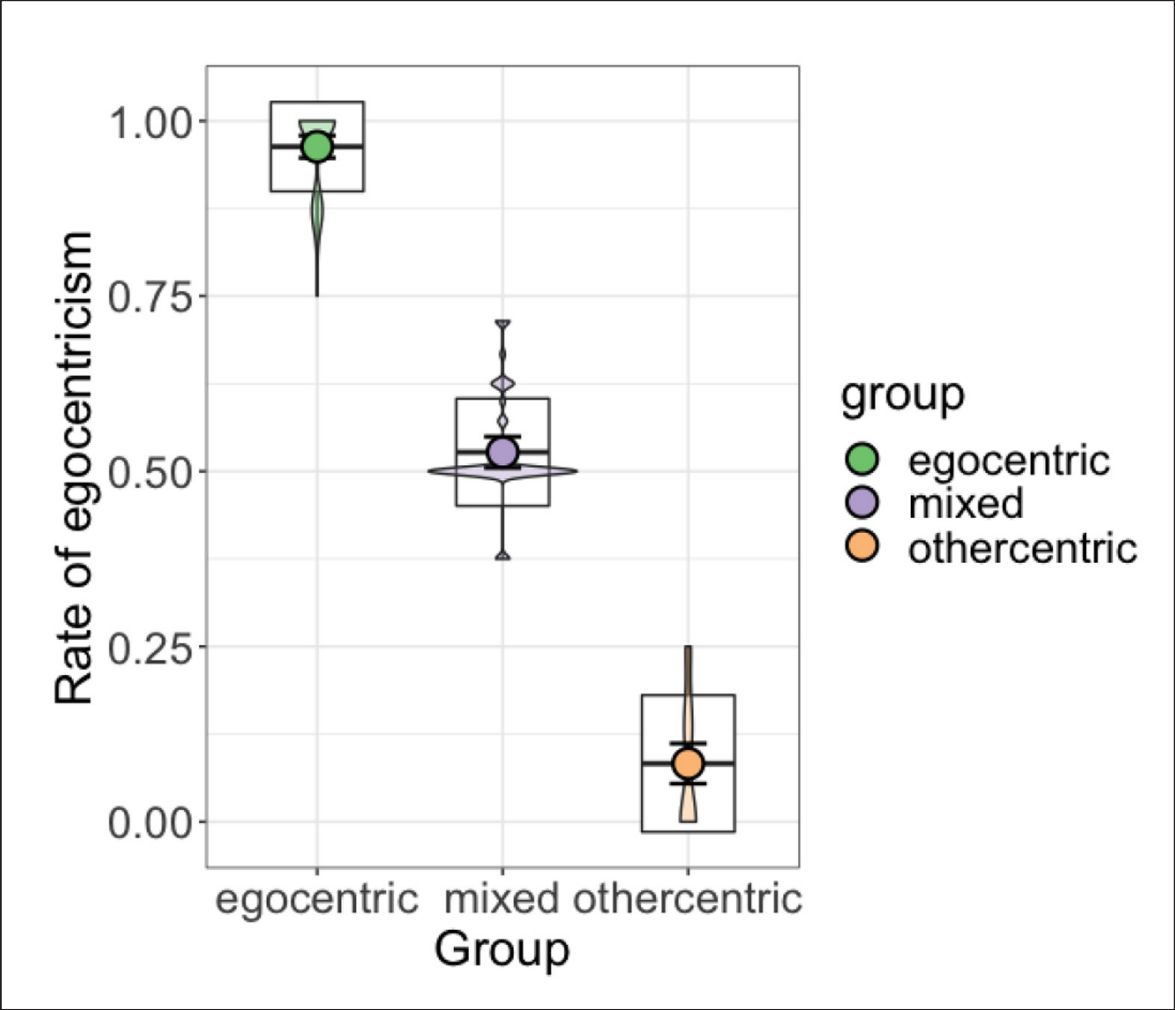
Mean rate of egocentricism for each group in the final three-group model. Boxplots represent ±1 standard deviation. Violin plots show data density.

The classification indices and fit statistics hence suggest both the three- and five-group models to be plausible representations of the data. To further explore this result, we took a closer look at the interpretability of the groups proposed by the models. The three-group solution classified participants into relatively even-sized clusters which can be described as ‘egocentric’, ‘mixed’, and ‘othercentric’ based on participants’ rate of egocentricism; the five-group solution proposed two additional intermediate groups: ‘mixed (more egocentric)’ and ‘mixed (more othercentric)’ (see Table 2). An examination of this solution taking into account the form of the partner’s instruction reveals that these clusters were primarily motivated by differences in right/left vs. front/back perspective taking tendencies. As can be seen from Figure 4, the ‘mixed (more egocentric)’ group consisted of participants who were largely egocentric, with egocentric behaviour occurring predominantly on right/left trials; while the ‘mixed (more othercentric)’ group were largely othercentric, and only occasionally egocentric on either right/left or front/back trials. We return to this result in the discussion. Although the five-group solution presents a more fine-grained picture of participants’ behaviour, the unbalanced distribution of participants across groups and less transparent interpretability of intermediate groups make it a poorer candidate for further analyses; hence, we opted to use the three-group solution for follow-up analyses including individual differences. Figure 5 shows the mean and standard deviation for each group in the three-group model.

### Individual differences in spatial perspective taking tendencies

To quantify the contribution of our three individual difference measures AQ_ss+c_ score, OPT deviation score, Stroop difference score) on participants’ egocentric behaviour, we used logistic mixed effects regression to model the dependent variable of whether or not participants selected the object from their own avatar’s perspective on each trial. Of interest here is participants’ behaviour on different perspective trials, where this response reflects egocentric perspective taking, as well as whether the effect of perspective condition is modulated by any of our individual difference measures. The model included perspective and all three individual difference measures as predictors, with each measure allowed to interact with perspective. Age and gender were also added as co-variates by including their respective interactions with perspective. Perspective was sum-coded (levels –0.5 for same and +0.5 for different), and individual difference measures were entered as scaled and centred continuous variables (z-scores). The model included by-participant and by-item random intercepts and by-participant random slopes for perspective. Model *R*^2^ values were obtained using the r.squaredGLMM function in the MuMIn package (Bartoń, 2023), which calculates conditional *R*^2^ by taking the sum of the variance of the fixed and random effects, over the sum of the variance of the fixed and random effects and the observational-level variance. We report beta coefficients, *p* values, and 95% confidence intervals for all significant effects in the text. The full model output for all (including non-significant) predictors is provided in Appendix A (Table 3), along with descriptive statistics on the mean, standard deviation, and distribution associated with each of our three individual difference measures.

**Figure 6:**
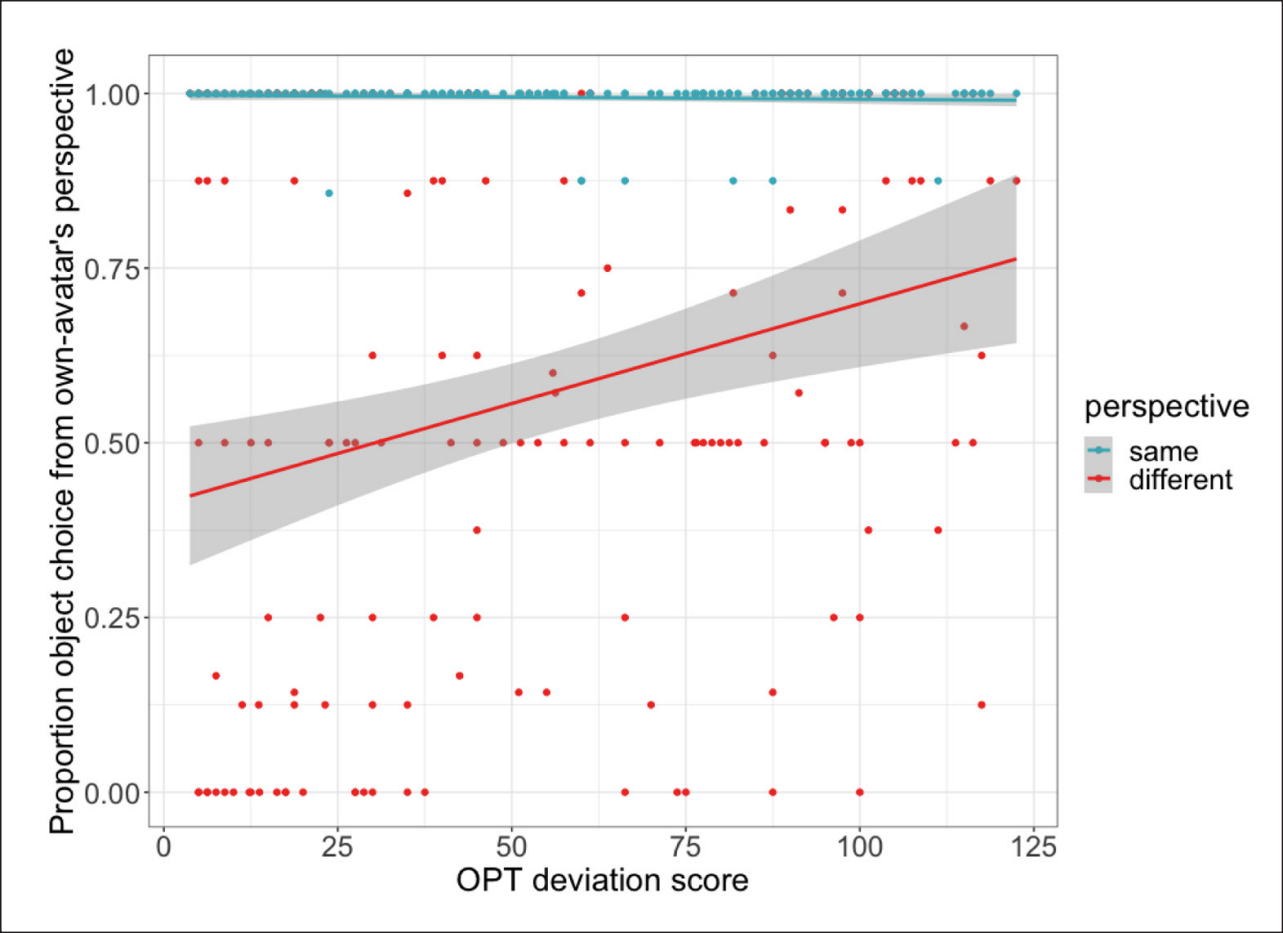
Relationship between participants’ OPT deviation score and the proportion of trials on which they selected the object from their own avatar’s perspective. On same perspective trials this was a shared perspective with the partner’s avatar; on different perspective trials this was across the table from the partner’s avatar and therefore reflects egocentric perspective taking. Higher OPT scores indicate poorer spatial orientation ability. Grey ribbons show 95% confidence intervals. Dots represent individual participants’ mean by perspective condition.

Our model showed an effect of perspective, with participants less likely to interpret their partner’s instruction egocentrically on different perspective trials, *β* = –4.59, *p* < .001, *CI* [–5.54, –3.64]. There was also an interaction between perspective and OPT deviation score, *β =* 1.21, *p* = .01, *CI* [0.29, 2.13], driven by a positive relationship between OPT score and participants’ egocentric rates, in particular on different perspective trials (see Figure 6). This was confirmed by separate analyses on each perspective condition, which showed that higher OPT deviation scores were associated with more egocentric perspective taking on different perspective trials, *β =* 0.82, *p* = .001, *CI* [0.32, 1.32], whereas no corresponding relationship was observed on same perspective trials (*p* > .3). This relationship is illustrated in Figure 6, which shows that on different perspective trials, participants’ object selection from their own-avatar’s perspective (i.e. rate of egocentricism) increases with increasing OPT deviation score (i.e. poorer spatial orientation ability). AQ_ss+c_ score, Stroop difference score, age, and gender were all not found to modulate perspective taking tendencies (all *p* > .4).

Our AQ_ss+c_ score was derived based on the social skills and communication subscales; however, some studies have found a correlation between other AQ subscales (e.g., imagination) and spatial perspective taking (Muto, 2021). Thus, we conducted an exploratory analysis in which we replaced our AQ_ss+c_ measure with participants’ scores for each individual subscale in turn. None of the individual subscales were found to significantly modulate perspective taking behaviour (see Appendix A Table 4), although the model with switching showed a marginally significant interaction between perspective and switching, *β =* –0.93, *p* = .07, *CI*[–1.94, 0.06]: higher switching scores (i.e. poorer switching ability, as measured by the AQ) were marginally more likely to be associated with less egocentric perspective taking on different perspective trials.^4^

To verify the unique contribution of OPT score on egocentric perspective taking behaviour, we constructed a final model including only the predictors of perspective and its interaction with OPT score. This model showed an effect of perspective, *β* = –4.70, *p* < .001, *CI* [–5.58, –3.81], and a significant interaction, *β* = 1.47, *p* < .001, *CI* [0.69, 2.26]. The model had a conditional *R*^2^ of 0.75, and was a significantly better fit than a reduced model that included only perspective, χ^2^(1) = 12.90, *p* < .001.

### Differences between latent groups

**Figure 7:**
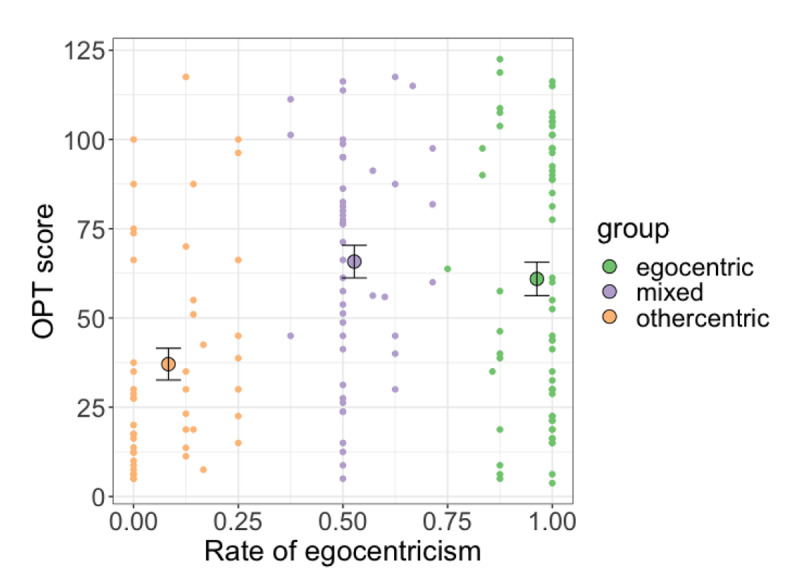
Mean rate of egocentricism and OPT deviation score for the three latent groups. Error bars represent ±1 standard error of group OPT score means. Dots represent individual participant points.

**Figure 8:**
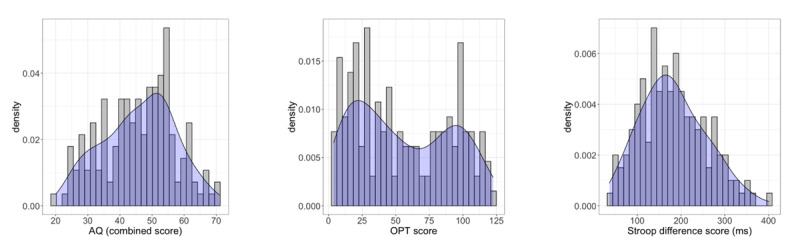
Distribution and density plots for participants’ AQ (combined), OPT, and Stroop difference scores.

Our final analysis focused on examining whether there were significant differences between the latent groups in their individual differences measures. This allowed us to evaluate whether the relationship between individual differences and spatial perspective taking was borne out in the latent groups we identified. We focused on the measure of OPT score, which was found to significantly modulate participants’ perspective taking behaviour. Figure 7 shows the mean OPT score for the three latent groups.

Linear regression was used to model participants’ OPT scores using latent group as a predictor. Group was coded with forwards Helmert coding, which compares each level of a predictor to the mean of subsequent levels. Based on our data which suggests that the difference lay in othercentric responders, we set the order of levels to be ‘othercentric’, ‘egocentric’, and ‘mixed’. This results in two comparisons: the first tells us whether there are any differences between othercentric responders and the combined group of egocentric and mixed responders; the second tells us whether there are any differences between egocentric and mixed responders. The model showed an effect of responder group for the first comparison: othercentric responders had lower OPT scores compared to egocentric and mixed responders, *β* = –26.29, *p* < .001, *CI* [–39.95, –14.63]. The second comparison showed no difference between the OPT scores of egocentric and mixed responders (*p* > .4).

## Discussion

The current study investigated individual differences in listeners’ spatial perspective taking behaviour. Participants completed a perspective taking task in which spatially ambiguous utterances produced by their partner could be interpreted egocentrically or othercentrically, followed by an individual differences battery targetting their spatial orientation ability, inhibitory control, and social preferences. The aim was to determine (a) whether we could identify latent groups of participants who differed in their spatial perspective taking behaviour, (b) which individual differences measures contributed to spatial perspective taking behaviour, and (c) how latent groups differed in these individual differences measures. Below, we address each of the three questions in turn, and discuss our findings in relation to the literature on individual differences and spatial perspective taking.

### Identification of latent participant groups

Our results demonstrate that it is possible to identify latent groups of participants based on their spatial perspective taking tendencies. Importantly, our groups were determined via a data-driven method of classification which compared clustering solutions of differing group sizes, rather than using pre-defined cut-off values, which may be determined post-hoc and simplify the actual picture of the data. The results from our LPA proposed both three and five groups to be plausible representations of the data. For the three-group solution, perspective taking tendencies are relatively comparable to Duran et al.’s three groups of ‘egocentric’, ‘othercentric’, and ‘mixed’ responders. Notably, our distribution of participants is roughly even across the three groups (see Table 2), in contrast to Duran et al. who observed a bimodal distribution of egocentric and othercentric responders and a comparatively smaller proportion of mixed responders. This may be partially due to the cut-off criteria for group membership determined by the LPA: ‘mixed’ responders, as posited by the three-group model, comprised participants whose rate of egocentricism fell between 37.5–75%, which is a slightly higher range than Duran et al.’s range of 30–70%. Crucially, however, the finding of stable participant groups supports a common observation in the broader literature on spatial cognition that individuals have systematic and consistent preferences for adopting a particular spatial perspective (Arnold, Spence, & Auvray, 2016; Beller et al., 2016; Job et al., 2021).

Further analysis of the five-group solution revealed that the additional clusters could be attributed to differences in response to the form of the partner’s instruction. Here, mixed participants appear to fall into three sub-groups, with differences tied directly to their behaviour on front/back vs. right/left trials: in particular, egocentricism is relatively low on front/back trials across all three mixed groups, but shows a decreasing trend from the ‘mixed (more egocentric)’ to the ‘mixed (more othercentric)’ group on right/left trials (see Figure 4). In other words, listeners are largely othercentric on front/back trials, but show greater variability on right/left trials. The fact that listeners are on the whole more othercentric on front/back trials aligns with findings from the spatial perspective taking literature, which demonstrate greater difficulty with right/left discrimination compared to other body-oriented directions (e.g., front/back or near/far; (e.g., front/back or near/far; Duran et al., 2011; Franklin & Tversky, 1990; Newcombe & Huttenlocher, 1992). Indeed, right–left confusion, in which people find it hard to distinguish right from left, is a well-established phenomenon in the spatial cognition literature (Wolf, 1973). While it was not our aim to investigate differences based on the form of instruction, these results demonstrate that the cognitive work involved in perspective taking is not equal across dimensions: in particular, right/left appears to pose a greater challenge than front/back (cf. Loy & Demberg, 2022). This in turn impacts listeners’ willingness to take their partner’s perspective. Beyond adding to the literature which demonstrates a right/left disadvantage in spatial reasoning, these results have implications for real-world perspective taking, in which people may be faced with tasks that involve discrimination on different dimensions (e.g., when giving directions remotely), and call for work to examine how othercentric tendencies differ across spatial dimensions and in response to combinations of spatial terms.

Interestingly, Muto et al. (2019) investigated spatial perspective taking in a task of judging directions relative to an avatar (right/left and in front of/behind), and found that the symmetry of the reference object, more so than the dimension of judgement, contributed to perspective taking difficulty. In particular, the typical right/left difficulty appeared diminished, and fewer people reported using ‘embodied rotation’ strategies, when the reference object was asymmetrical on the right/left plane (e.g., a chair with only one armrest). Conversely, in front of/behind judgements appeared more difficult when the reference object was front/back symmetrical, highlighting that the front/back advantage observed in the literature may be an artefact of the front/back asymmetric objects typically used in studies (e.g., avatars with faces that serve as visual cues). While we do not observe similar results to Muto et al., we highlight some differences between their study and ours. Firstly, they investigated judgements for objects ‘in front of’ or ‘behind’ a reference object (also known as level-1 spatial perspective taking). This is theorised to rely on processes similar to level-1 visual perspective taking, which does not necessitate an embodied rotation into a target perspective, and can be solved using visual cues to determine the reference’s front/back side (Surtees et al., 2013a). Our study, on the other hand, investigated judgements for objects in the ‘front’ or ‘back’ from a reference perspective. Notably, both of these still lie in front of the reference perspective (differing just in terms of distance), and therefore visual cues, such as a face on the avatar, would be less useful. In that regard, our front/back perspective task likely still elicited an embodied perspective rotation (i.e. a level-2 spatial perspective problem), despite being in the front/back dimension. This is supported by our finding that listeners’ spatial orientation ability was related to their spatial perspective taking tendencies in the task, and suggests that the front/back and right/left distinction proposed by Surtees et al. (2013a) to differentiate between processes at play may be too simplistic. Muto et al. (2019) also manipulated the reference object’s symmetry plane, whereas our study made use of two-dimensional avatars that were right-left symmetrical with no distinguishable front or back side. Here, they found that whether the reference object was symmetrical or not on the front/back axis affected the strategies employed for in front of/behind judgements. While we speculate that this distinction may be less relevant in a front/back judgement task such as ours, our design does not allow us to preclude its relevance (for instance it is conceivable that having an intrinsic ‘front’ side may allow listeners to more easily identify objects that are closer to (front) and farther from (back) the avatar). The role of reference symmetry in front/back perspective judgements would be a useful avenue for future work to pursue.

### Contribution of individual differences

Having established that listeners differ in their spatial perspective taking tendencies, we sought to identify which factors contribute to this variability. We found that listeners’ egocentric tendencies in the main task were related to their spatial orientation ability, with poorer spatial orientation ability linked to more egocentric perspective taking. These results have theoretical relevance for the question of the processes underlying spatial perspective taking. In particular, our results support the view that spatial perspective taking is an embodied cognitive transformation involving a mental update of one’s ‘self’ representation relative to the environment. As such, spatial orientation ability appears to play a key role in this process of self-reorientation.

Several studies that have used the Object Perspective Test as a measure of individual differences demonstrate a relationship between spatial orientation ability and tasks of spatial reasoning. For instance, better spatial orientation is linked to better performance in route navigation or learning new environments (Schinazi et al., 2013; Weisberg et al., 2014). Notably, the tasks employed by these studies typically involve an element of instructed perspective taking (e.g., being told to estimate the distance between buildings, or the direction from one building to another in three-dimensional space). In the current study, we show that this relationship extends to spontaneous perspective choice; in other words, spatial orientation ability predicts not only performance in, but also motivation to engage in tasks of spatial reasoning. This is an important aspect of perspective taking, since many real-life situations do not involve being explicitly told to take another’s perspective; nevertheless, it is a process that is highly relevant in collaborative situations (e.g., Hayashi, 2018), and under some models of communication, allows us minimise collective effort and maximise mutual understanding (Clark, 1996; Clark & Wilkes-Gibbs, 1986). A further point of note is that much of previous research on instructed perspective taking has employed tasks that can be largely solved based on memory processes, for instance remembering a previously-seen array (Shelton & McNamara, 2004; R. F. Wang, 2005) or a previously-learned environment (Weisberg et al., 2014). Whilst such memory-based processes are no doubt useful in many perspective taking situations (e.g., giving directions), many day-to-day spatial problems in fact call upon real-time, in-the-moment processes such as whether, and how to, take a conversation partner’s perspective (Brown-Schmidt & Hanna, 2011; Schober, 1993).

One strength of the current study is that we explored the contribution of a range of individual differences, which have not typically been investigated together in the context of spatial perspective taking. Our results thus shed light on the nature of the processes underlying this cognitive operation. Although we do not provide a direct test of the relevant mechanism(s), it is noteworthy that our individual differences measures target largely distinct processes which have been separately attributed to spatial perspective taking. Here, we examined the relative contribution of these mediating factors within the same set of participants in a single task. We found an influence of spatial orientation ability, but no evidence for inhibitory control or social preferences, on listeners’ spatial perspective taking tendencies. This highlights the role of cognitive mechanisms, in particular spatial cognition, over other mechanisms such as socially-driven processes in spatial perspective taking. However, it is possible that our experimental context may have restricted the potential to observe an influence of these factors. The role of inhibitory control, for instance, has been primarily noted in developmental and non-neurotypical populations (Frick & Baumeler, 2017; Kuijper, Hartman, & Hendriks, 2021). Healthy young adults such as our participants, on the other hand, may as a group already be functioning at a level beyond that called for in perspective taking. Inhibition effects also tend to be largely associated with visual perspective taking, whereas evidence for its role in spatial perspective taking, as noted earlier, is more limited. Visual perspective taking studies showing an effect of inhibition have typically couched their findings in terms of activation, in that inhibition-control processes are called upon to reduce activation of salient visual or linguistic competitors (Brown-Schmidt, 2009; Wardlow, 2013). While it is reasonable to assume some level of commonality in inhibitory processes across different domains (Apšvalka, Ferreira, Schmitz, Rowe, & Anderson, 2022), it is possible that suppressing salient visual input is not entirely similar to suppressing a conceptual representation of one’s body schema. In that regard, our results speak to the argument for some degree of difference in the mechanisms employed by the two processes. Notably, spatial perspective judgements have been shown to make use of embodied self rotations to a greater extent than visual perspective judgements (Surtees et al., 2013b). This is in accordance with our finding that participants’ performance in the OPT, which makes use of embodied perspective transformations, was a significant predictor of their spatial perspective taking tendencies in the current task.

Perhaps more surprisingly, we saw no evidence for a mediating role of social preferences, which has been implicated in several earlier studies (Clements-Stephens et al., 2013; Job et al., 2021; Shelton et al., 2012). Shelton et al. (2012), for instance, similarly tested the role of multiple individual differences on spatial perspective taking. They found a significant contribution of social skills (using a similar measure of AQ_ss+c_) and object rotation ability (as measured by the Vandenberg Mental Rotation Test), although social skills accounted for a much larger share of the variance (34%) than object rotation (5%) in the regression. However, we note several critical differences between their study and ours, namely their use of an instructed perspective taking paradigm, and the fact that they did not include a test of spatial orientation ability. Object rotation and spatial orientation are known to rely on distinct cognitive processes (Hegarty & Waller, 2004), with the latter being a strong predictor of performance in tasks of spatial cognition (e.g., Weisberg et al., 2014); including a test of spatial orientation would thus provide a more holistic picture of the factors contributing to spatial perspective taking.

Shelton et al. (2012) also noted that the significant contribution of social skills in their study occurred only in the condition utilising a potentially agentive target (a wooden doll), and not with non-agentive targets (a camera or a wooden block). This suggests that social preferences may be relevant only when the social element of a spatial task is explicit enough. Our use of a computer partner in our task likely downplayed the socially-relevant aspects of the interaction, thus minimising the likelihood that social preferences would come into play. Xiao et al. (2021) found a relationship between speakers’ social skills and their othercentric tendencies in a spatial description task, but only in participants who were told they were addressing a human and not a robot, and venture that speakers regard humans, but not robots, as social partners. More generally, we note that this pattern of results may be a reflection of peoples’ expectations towards a computer’s technological, rather than interpersonal capabilities; that is, perspective taking may be regarded as a performance-based rather than socially-oriented function in computers. As a result, listeners may have specific expectations about a computer’s perspective taking abilities, which may be orthogonal to the social bearings of perspective taking in interaction. Qualitative interviews on peoples’ preferences and expectations regarding robot capabilities underscore this dissociation: these reveal that people largely expect robots to help with work-related activities (e.g., household chores, information management) but less so with socially-relevant tasks (e.g., entertaining guests), and to refrain from exhibiting human-like social behaviour such as emotions or intentions (Horstmann & Krämer, 2019; Smarr et al., 2014). When asked to evaluate robot characteristics, respondents also tended to visualise robots as performance-driven machines (e.g., being efficient and precise), rather than social devices (e.g., being friendly, having emotions; Ezer, Fisk, & Rogers, 2009). This may suggest a delineation that people draw between robot and human interactional partners, and their different expectations regarding the technological and social capabilities of a machine.

### Differences between latent groups

Our final question asked how the latent groups we observed in our first analysis differed in their individual differences. This allows us to go beyond simply identifying trends of mediating variables in our data, to mapping where exactly differences lie between subgroups of participants. We focused on the three-group solution since this model has more readily interpretable groups. Here, we found significant differences in OPT scores across the three groups. In particular, the group of othercentric responders stood out amongst participants as having better spatial orientation ability; egocentric and mixed responders, on the other hand, were no different in this measure. These results are significant on two fronts. Firstly, we show that there are measurable differences between the latent groups of participants in our data, and that these differences lie in participants who are consistently inclined to take their partner’s perspective. Our finding that poorer spatial orientation ability was associated with greater egocentricism also highlights the cognitive aspect of spatial perspective taking. Accordingly, participants who found it more challenging to reorient themselves mentally appeared less willing to take their partner’s perspective. Given the simplicity of our paradigm, another approach could have been for listeners to adopt a simple heuristic of choosing the object ‘opposite’ to that of their own perspective on different perspective trials. However, the fact that their perspective taking tendencies were related to their spatial orientation ability suggests that this was not the case. Rather, listeners seemed to invest in the mental effort of reorienting themselves with their partner’s perspective, with those who found this task more cognitively demanding being less motivated to do so. Secondly, the fact that egocentric and mixed responders did not differ in their OPT scores highlights that they performed similarly in this task. While one might expect differences to emerge across all three groups, it is worth remembering that the rate of egocentricism for mixed responders posited by our three-group model is slightly skewed towards egocentricism (37.5%–75%); thus, as a group, mixed responders are already closer to egocentric responders with respect to their perspective taking tendencies. In light of that, it is perhaps less surprising that their OPT scores are also similar. Crucially, however, our results highlight that it is the othercentric group that stands out from the other two groups. This suggests that it is specifically the act of taking another’s perspective that is being captured by differences in OPT scores here. Thus, although our data-driven classification identifies three groups of participants who differ in their egocentric tendencies, where crucial differences with regard to their underlying cognitive profile lie appear to be the predominantly othercentric listeners.

## Conclusion

The present results show that listeners differ in their tendencies to take a partner’s spatial perspective. This variability manifests in stable subgroups of participants who are consistent in their behaviour, and this behaviour correlates with differences in participants’ spatial orientation ability. An open question is how stable these perspective taking tendencies are over longer periods of time. Our data only speak to the fact that individuals show consistent tendencies within a single experiment session; however, there is evidence that people exhibit consistency over longer periods in a range of other phenomena, such as their linguistic production, moral reasoning, or pro-social behaviour (de Boer, Quené, & Heeren, 2022; Eisenberg et al., 2002; Puntiroli, Moussaoui, & Bezençon, 2022). Whether similar longitudinal effects are seen in perspective taking tendencies remains a question for future research to examine. At present, our results provide insight on the nature of the mechanism underlying perspective taking, and support the view that spatial perspective taking involves a mental transformation of one’s self-representation relative to the environment. As such, spatial perspective taking can be seen as a process of embodied cognition in which people disengage from their own position in space to take on a different, imagined position; people who are better able to perform this cognitive operation are more likely to adopt an othercentric perspective. Importantly, unlike tasks of instructed spatial perspective taking which have been used by many previous studies, this relationship was observed in the context of perspective choice, in which both egocentric and othercentric perspectives were valid and listeners were free to adopt either. Thus, for listeners who have sufficient resources, the mental effort of re-orientation is something that they are willing to invest in even when the situation does not specifically call for it.

## Data Accessibility Statement

All data and analysis scripts for this study are available on the Open Science Framework (OSF) repository: https://osf.io/9unv8/?viewonly=83717d835c2749faac0b8e5d3089b3aa.

## Figures and Tables

**Figure:**
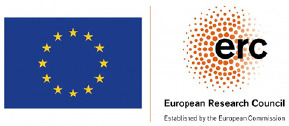


## Footnotes

In addition, based on previous findings which suggest that right/left perspective taking is more effortful than front/back (Duran et al., 2011; Mainwaring, Tversky, Ohgishi, & Schiano, 2003), we implemented a novel task of direction discrimination in which participants responded to spoken instructions in the right–left and front–back dimensions. However, due to issues resulting from data loss this task was ultimately omitted from analyses. For completeness, we report the task procedure in Appendix B.

www.mturk.com.

We note that our results do not change if we include this subset of participants.

This is in the opposite direction to what we might expect given findings to suggest an association between poorer social/communication ability (as measured by the AQ) and more egocentricism (e.g. Shelton et al., 2012). However, we refrain from over-interpreting it since the relationship between switching and spatial perspective taking is less clear, and the effect becomes non-significant if we apply a multiple comparisons correction for analysing each subscale individually.

## Appendix A

**Table 3:** Full model output including perspective, all three individual difference measures, and age and gender on the outcome variable of whether participants selected the object from their own avatar’s perspective. On different perspective trials this reflects whether participants took their own or their partner’s spatial perspective. Individual difference measures and age were scaled and centred by converting to z-scores. Perspective and gender were sum-coded (perspective: same –0.5, different +0.5; gender: female –0.5, male +0.5).

	*β*	*Z*	*SE*	*P*	95% *CI*

(Intercept)	3.01	0.24	12.30	<.001	[2.53, 3.49]

perspective	–4.59	0.48	–9.49	<.001	[–5.54, –3.64]

AQ_ss+c_	–0.10	0.23	–0.40	.69	[–0.55, 0.37]

OPT score	–0.20	0.24	–0.84	.40	[–0.27, 0.67]

Stroop difference	–0.02	0.22	–0.08	.94	[–0.45, 0.42]

perspective:AQ_ss+c_	–0.23	0.46	–0.49	.62	[–1.13, 0.67]

perspective:OPT score	1.21	0.47	2.58	.01	[0.29, 2.13]

perspective:Stroop difference	0.45	0.43	1.06	.29	[–0.39, 1.30]

perspective:age	0.39	0.39	1.00	.31	[–0.38, 1.16]

perspective:gender	–0.08	0.84	–0.09	0.93	[–1.56, 1.72]

**Table 4:** Exploratory analyses with individual AQ subscales and the full AQ: Perspective by AQ (subscale) interaction results for each model. Note that these were run as separate linear mixed effects regression models in which we replaced our AQ_ss+c_ measure with each subscale measure in turn.

MODEL WITH	*β*	*Z*	*SE*	*P*	95% *CI*

AQ (communication)	0.21	0.47	0.45	.65	[–0.72, 1.14]

AQ (detail)	–0.15	0.44	–0.33	.74	[–1.01, 0.71]

AQ (imagination)	–0.52	0.50	–1.04	.30	[–1.51, 0.46]

AQ (social)	–0.39	0.35	–1.12	.26	[–1.08, 0.29]

AQ (switching)	–0.93	0.51	–1.84	.07	[–1.94, 0.06]

AQ (full)	–0.19	0.24	–0.77	.44	[–0.65, 0.29]

### Individual differences data summary

**Table 5:** Mean, standard deviation, minimum, and maximum scores for individual differences measures used in the study.

	MEAN	SD	MIN	MAX

AQ_ss+c_	45.8	11.3	20	71

OPT	55.4	35.7	3.8	122.5

Stroop	182.5	75.1	36.4	400.6

*Notes*: AQ = Autism Quotient (combined score derived from social+communication subscales); OPT = Object Perspective Task; Stroop = Stroop difference score (mean incongruent – congruent RT).

**Table 6:** Correlation between individual difference measures used in the study.

	AQ_SS+C_	OPT	STROOP

AQ_ss+c_	1.00	–	–

OPT	–0.22	1.00	–

Stroop	0.15	0.02	1.00

**Table 7:** Mean, standard deviation, minimum, and maximum scores for individual AQ subscales and the full AQ.

SUBSCALE	MEAN	SD	MIN	MAX

Communication	20.7	5.2	10	33

Detail	23.7	5.3	12	39

Imagination	20.6	4.7	11	34

Social	25.2	7.0	10	39

Switching	26.5	5.0	15	38

Full AQ	116.9	19.2	72	177

**Table 8:** Correlations between AQ subscale scores.

	COMMUNICATION	DETAIL	IMAGINATION	SOCIAL	SWITCHING

Communication	1.00	–	–	–	–

Detail	0.13	1.00	–	–	–

Imagination	0.49	0.15	1.00	–	–

Social	0.70	0.00	0.55	1.00	–

Switching	0.56	0.04	0.35	0.61	1.00

## Appendix B

### Individual differences task: Direction discrimination ability

Based on previous findings that perspective taking tendencies might be related to the relative difficulty with different dimensions of spatial ambiguity (e.g., right/left vs. front/back; cf. Duran et al., 2011; Mainwaring et al., 2003), we implemented a novel task of direction discrimination in which participants responded to spoken instructions in the right–left and front–back dimensions. The aim of the task was to explore whether greater difficulty with right–left discrimination would be related to poorer perspective taking. However, due to data loss from issues writing data to the server as well as some participants misinterpreting the task instructions, the low number of usable participants substantially reduced power for this test. Here, we summarise the task setup and procedure; however, since our analyses required participants to have completed the full set of tasks, the data from this task were ultimately omitted from analyses.

The task was designed to assess participants’ ability to quickly recognise and respond to directions in the right–left and front–back axes. In the task, participants had to respond as quickly as possible to an instruction to click on one of four identical moles on the screen. The moles were arranged in a similar configuration to the objects in the main experimental task – front, back, left, and right, as viewed from the perspective of the participant’s avatar located at the bottom of the screen. Between trials, participants clicked a ‘ready’ button to reset their cursor to the centre position, after which the mole images appeared. Playback of the audio instruction began after a variable delay of 1,500—5,000 ms. Participants were told to respond as quickly and accurately as possible; trials timed out automatically after 4s if a click on a mole was not registered. Participants completed 32 trials (eight each of front, back, left, and right), presented in random order with the constraint that no two consecutive trials targeted the same position. A difference score for each participant was calculated by taking the difference between their mean response time on right/left trials and front/back trials, with larger difference scores reflective of greater difficulty with right–left relative to front–back discrimination.
